# Gas Permeability and Mechanical Properties of Polyurethane-Based Membranes for Blood Oxygenators

**DOI:** 10.3390/membranes12090826

**Published:** 2022-08-24

**Authors:** Inês Coelho, Rita F. Pires, Sérgio B. Gonçalves, Vasco D. B. Bonifácio, Mónica Faria

**Affiliations:** 1 Center of Physics and Engineering of Advanced Materials (CeFEMA), Laboratory for Physics of Materials and Emerging Technologies (LaPMET), Chemical Engineering Department, Instituto Superior Técnico, Universidade de Lisboa, Av. Rovisco Pais, 1049-001 Lisbon, Portugal; 2 IDMEC, Instituto Superior Técnico, Universidade de Lisboa, Av. Rovisco Pais, 1049-001 Lisbon, Portugal; 3 iBB-Institute for Bioengineering and Biosciences and i4HB-Institute for Health and Bioeconomy, Instituto Superior Técnico, Universidade de Lisboa, Av. Rovisco Pais, 1049-001 Lisbon, Portugal; 4 Bioengeneering Department, Instituto Superior Técnico, Universidade de Lisboa, Av. Rovisco Pais, 1049-001 Lisbon, Portugal

**Keywords:** polyurethane membrane, blood oxygenator, gas permeation, solution-diffusion, phase segregation, Young’s modulus, tangent modulus, yield strength, ultimate tensile strength, rupture point

## Abstract

The production of medical devices follows strict guidelines where bio- and hemocompatibility, mechanical strength, and tear resistance are important features. Segmented polyurethanes (PUs) are an important class of polymers that fulfill many of these requirements, thus justifying the investigation of novel derivatives with enhanced properties, such as modulated carbon dioxide and oxygen permeability. In this work, three segmented polyurethane-based membranes, containing blocks of hard segments (HSs) dispersed in a matrix of soft segment (SS) blocks, were prepared by reacting a PU prepolymer (PUR) with tris(hydroxymethyl)aminomethane (TRIS), Congo red (CR) and methyl-β-cyclodextrin (MBCD), rendering PU/TRIS, PU/CR and PU/MBCD membranes. The pure (control) PU membrane exhibited the highest degree of phase segregation between HSs and SSs followed by PU/TRIS and PU/MBCD membranes, and the PU/CR membrane displayed the highest degree of mixing. Pure PU and PU/CR membranes exhibited the highest and lowest values of Young’s modulus, tangent moduli and ultimate tensile strength, respectively, suggesting that the introduction of CR increases molecular mobility, thus reducing stiffness. The CO_2_ permeability was highest for the PU/CR membrane, 347 Barrer, and lowest for the pure PU membrane, 278 Barrer, suggesting that a higher degree of mixing between HSs and SSs leads to higher CO_2_ permeation rates. The permeability of O_2_ was similar for all membranes, but ca. 10-fold lower than the CO_2_ permeability.

## 1. Introduction

Segmented polyurethanes (PUs) are a class of polymers with widespread use in medical devices because of their good bio- and hemocompatibility, mechanical strength, tear resistance, and versatility in tailoring the final bulk and surface properties [1]. Most segmented PUs are composed of a polyether, polyester, or polybutadiene amorphous soft segment (SS), which confers an elastomeric character, and a polyurethane/urea crystalline, or glassy hard segment (HS), which confers mechanical strength.

PUs are attractive materials for the preparation of membranes since they exhibit a variety of physical and chemical properties, depending on the reagents and the synthesis conditions, and can be easily produced at a commercial scale. Gas permeation membranes prepared by segmented PUs is well documented [2,3,4,5,6,7] and it is known that their properties are greatly influenced by the microphase-separation degree (degree of phase separation between soft and hard segments) [8,9].

**Figure 1:**
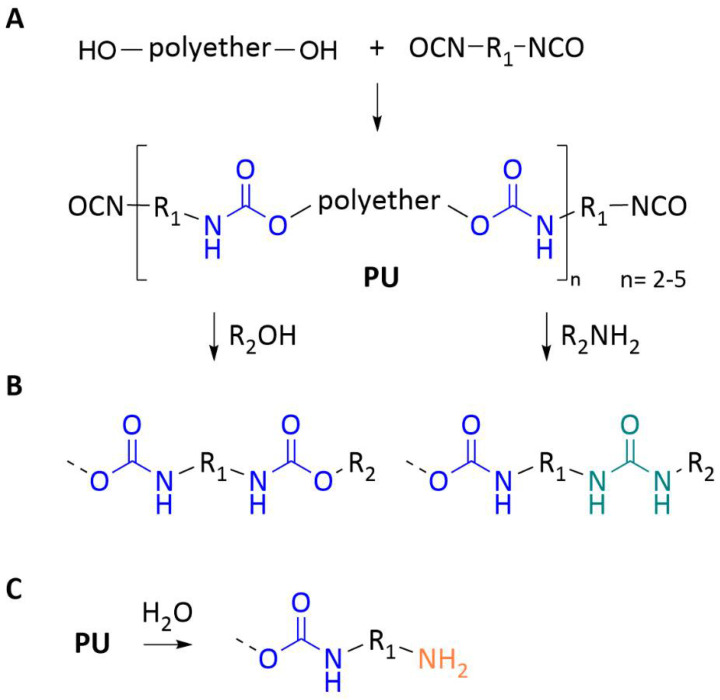
General scheme for the preparation of a PU prepolymer from macrodiol and diisocynate reagents (**A**) and the corresponding products from the reaction with alcohols and amines (**B**) and hydrolysis in the presence of water (**C**).

Segmented PUs can be prepared from PU prepolymers (often referred to as resins or oligomers) which are, in general, high-molecular-weight, high-viscosity liquids that contain highly reactive isocyanate (NCO) groups. The first step in PU prepolymer synthesis is the reaction of a diisocyanate with a selected macrodiol (e.g., polyether or polyester diols), leading to the formation of urethanes (-NHCO_2_), as illustrated in Figure 1A. The use of an excess of the diisocyanate component ensure that the resulting materials are end-capped with isocyanate groups for further reactions. Usually, this prepolymer has a degree of polymerization between 2 and 5.

The prepolymer terminal NCO groups can further undergo addition reactions with selected reagents such as alcohols and amines, forming urethanes and ureas (NH-CO-NH), respectively (Figure 1B). The reaction with water results in unstable carbamic acids that decompose to the corresponding amines with release of carbon dioxide (Figure 1C). After undergoing the different addition reactions, any unreacted isocyanate groups interact with air moisture and turn into amines. 

Isocyanate terminated prepolymers can be synthesized with varying chemical structures, molecular weights, number of isocyanate functional groups and spatial arrangements, leading to a variety of polymer structures and consequently to PU-based membranes with different permeation characteristics.

Xiao et al. [10] studied the permeation of CO_2_ through PUs containing HSs derived from 4,4′-diphenylmethane diisocyanate chains and SSs composed of poly(propylene oxide) (PPO). They found that the permeabilities decreased with the increase in HS content varying from 88 Barrer to 13 Barrer for membranes with the HS content varying from 32% to 65%. Sadeghi et al. [6] studied the effect of urethane and urea content on the gas permeation properties of poly(urethane-urea) membranes containing 1,4 butanediol (BDO) as the SS and found that the CO_2_ permeability increased with the increase in SS content and hence the decrease in HS content. CO_2_ permeabilities varied from 93 to 119 Barrer when the SS content increased from 0 to 75% and hence the HS content decreased [6]. Diseases of the respiratory system are the third main cause of death in the EU, preceded only by circulatory diseases and various types of cancer. One of the forms of therapy being used in critical care for the management of severe respiratory and cardiac failure is Extracorporeal Membrane Oxygenation (ECMO). The main component of the ECMO circuit is the blood oxygenator (BO) composed of gas permeation membranes responsible for driving O_2_ into the blood from a sweep gas source and removing excess CO_2_ from the blood. To be efficient, blood oxygenators (BOs) must have high O_2_ and CO_2_ transfer rates and be hemocompatible.

Currently, over 98% of BOs are composed of microporous polypropylene (PP) or asymmetric polymethylpentene (PMP) hollow fiber membranes. PP membranes exhibit high gas permeation rates due to the negligible resistance offered by the membrane itself as O_2_ diffuses freely and quickly from the gas compartment through the membrane pores, and then into the blood. Despite complying with the gas exchange requirements, these BOs are often faced with the challenge of plasma leakage [11] and, although increasing the sweep gas pressure can counteract plasma leakage, it risks introducing gas emboli into the blood stream. Therefore, plasma wetting eventually requires replacing the BO and reconditioning the patient’s blood to the new foreign surface. Plasma leakage seldom occurs in PMP BOs due to the asymmetric nature of the membranes; however, insufficient hemocompatibility limits their use and compromises their longevity [12]. Upon contact with the membrane surfaces, fibrinogen deposition and consequent activation of coagulation factors and complement allow platelets and leucocytes to adhere to BO surfaces and enhance thrombin generation. Although systemic anticoagulants are administered during ECMO, clotting in the BO remains an issue [13], resulting in higher rates of thrombosis in ECMO patients. The limitations of both PP and PMP membranes require the replacement of the BO several times during prolonged ECMO treatment which calls for further patient anticoagulation increasing the risk of bleeding [11]. A recent clinical study revealed that over 50% of patients requiring prolonged respiratory support died during ECMO or in the next 60 days due to different complications, 30% of which were associated with oxygenator dysfunction requiring replacement [14].

Considering the enhanced hemocompatibility and easily tailored surface and mechanical properties associated with PU-based biomaterials, our research group has focused on the preparation of segmented PU membranes for blood oxygenators [15]. For this, a PPO-based polyurethane prepolymer (PUR) containing three isocyanate terminal groups with an average molecular weight (MW) of 3500 g/mol was used [16,17,18,19]. Figure S1 shows the molecular structure of the PUR prepolymer used in our studies. Dense symmetric PUR membranes were synthesized by the solvent evaporation method, where, during the curing process, NCO groups react with water from moisture. This hydrolysis leads to the possible formation of urethane and urea groups according to reactions shown in Figure 1B. Figure S2 shows the most probable molecular structure of the PUR membranes characterized by blocks of HSs composed of diisocyanate, urethane and urea groups which are dispersed in a matrix of SS blocks composed of a sequence of PPO molecules. In order to correlate polymer network structures with membrane permeation, the membranes were characterized in terms of phase segregation between HSs and SSs by attenuated total reflectance Fourier transform infrared spectroscopy (ATR-FTIR) as well as oxygen and carbon dioxide permeability [20]. Results showed the existence of hydrogen bonding between NH and C=O groups, suggesting the formation of HS aggregates dispersed throughout a SS matrix composed of PPO. Gas permeation studies revealed CO_2_ and O_2_ permeability coefficients of 188 and 10 Barrer, respectively [20]. While the CO_2_ permeability coefficient is in the range of values exhibited by PMP membranes used in current BOs (92 Barrer), the O_2_ permeability is below the desirable threshold (32 Barrer) [20,21] and further studies of membranes composed of the PUR polymer were needed to improve O_2_ permeation. In terms of hemocompatibility, the PUR membranes exhibited promising results as they were proved to be non-hemolytic and showed low thrombosis degrees [16].

The versatility of the structure design of polyurethane membranes can be further increased by the introduction of a second type of SS [22]. In these PUs, phase segregation can occur between SSs and HSs (similar to what occurs in PUs containing one type of SS) as well as different extents of phase separation between the two types of SSs opening up new possibilities for tuning bulk and surface membrane properties [23]. With the aim of further enhancing the hemocompatibility of the PUR membranes (containing one type of SS) as well as increasing the O_2_ permeability while maintaining high CO_2_ permeation rates, our research group focused on the synthesis of PU membranes containing two types of SSs. Two types of PU-based copolymer membranes containing PPO as the first SS were prepared: (i) PU/PBDO membranes containing poly(butadiene diol) (PBDO) as the second type of SS [24,25], and (ii) PU/PCL membranes containing polycaprolactone (PCL) as the second type of SS [16,17,18,19,20,26]. 

Copolymer PU/PBDO membranes were synthesized by reacting the same PUR prepolymer with PBDO with PUR/PBDO weight ratios of 80/20 and 33/67. This led to dense symmetric membranes composed of blocks of HSs similar to the ones found in the PUR membrane (formed by diisocyanate, urethane and urea groups) and blocks of SSs composed of PPO and PBDO molecules. The number of SSs in the copolymer PU/PBDO membranes is much higher than for the PUR membranes and increases as the PBDO content increases. Phase segregation between HSs and SSs was studied by ATR-FTIR and results showed the existence of hydrogen bonded HSs with formation of urethane/urea aggregates. The increase in PBDO content led to higher mixing between the two SSs as well as a decrease in aggregation between urethane/urea HSs [25]. Furthermore, the CO_2_ permeability of the PU/PBDO membranes was dependent on the PU:PBDO ratio and decreased with increase in PBDO content: the CO_2_ permeability of the PU/PBDO 80/20 and 33/67 membranes was 150 Barrer and 90 Barrer, respectively. For the PU/PBDO membranes, it was concluded that larger quantities of the second SS led to higher degrees of mixing between SS and HS microphases which resulted in lower CO_2_ permeabilities [24]. In terms of hemocompatibility, the PU/PBDO membranes exhibited very poor characteristics, namely in terms of high thrombosis degrees [4], and therefore no further studies were performed with these membranes.

Copolymer PU/PCL membranes with PUR/PCL ratios of 95/5 and 90/10 were synthesized by reacting the same PUR prepolymer with polycaprolactone diol (PCL-diol). ATR-FTIR analysis showed the presence of HS clusters in both membranes and that the aggregation of HSs is dependent on the ratio of the two SSs and increases with the increase in PCL content. It is concluded that the increase in PCL content reduces the freedom of the HSs allowing for fewer opportunities of their mixing with the SSs. In terms of gas permeation, it was found that CO_2_ permeability was 250 Barrer and 337 Barrer for the membranes containing 5 and 10 wt.% of PCL, respectively, indicating that PCO_2_ is higher in membranes where phase segregation between HSs and SSs is higher. The O_2_ permeability coefficients of the copolymer PUR/PCL membranes were similar to the value found for the pure PUR membrane (~10 Barrer) and did not vary with the PCL content [20]. In terms of hemocompatibility, the PU/PCL membranes revealed very good results, namely in terms of being non-hemolytic, negligible thrombosis degrees and low platelet adhesion and activation [16,17,18].

The production of membranes prepared by casting solutions composed of the PUR prepolymer and another component with highly reactive functional groups yields a great number of possibilities in the design of novel gas permeation membranes with polymer network structures of increased complexity.

With the aim of preparing novel PU-based membranes, using the PUR prepolymer, with higher O_2_ permeability while preserving the high CO_2_ permeability and hemocompatibility properties of membranes produced in the past, the present work addresses the synthesis of three different PU-based membranes by reacting the PUR prepolymer with: (i) tris(hydroxymethyl)aminomethane (TRIS), rendering the PU/TRIS membrane, (ii) Congo red (CR), rendering the PU/CR membrane, and (iii) methyl-β-cyclodextrin (MBCD), rendering the PU/MBDC membrane. Having amine and/or alcohol groups in their structure, these molecules reacted easily with the PUR prepolymer to produce novel segmented PU-based membranes. Furthermore, the presence of these functional groups have proved to enhance the permeation of O_2_ and/or CO_2_ [27,28,29]. The reaction of PUR with an aliphatic amine (TRIS), an aromatic dye (CR) or a cyclodextrin, composed of sugar molecules arranged in a cycle with a well-defined cavity (MBCD) led to novel PU-based membranes with distinct properties. ATR-FTIR studies were conducted to characterize the membranes in terms of phase segregation between SSs and HSs, O_2_ and CO_2_ gas permeability rates were measured by the constant volume method and the mechanical properties were studied by evaluating the differences in the principal tensile properties (Young’s modulus, tangent modulus, yield strength, ultimate tensile strength and rupture point). Finally, the chemical structure of the membranes was correlated to the gas permeation and mechanical properties.

## 2. Materials and Methods

### 2.1. Materials

The poly(propylene oxide) (PPO)-based polyurethane prepolymer (PUR) containing three isocyanate terminal groups with an average molecular weight (MW) of 3500 g/mol was supplied by Fabrires—Produtos Químicos S.A., Lisbon, Portugal. Tris(hydroxymethyl)aminomethane (TRIS) (purity ≥ 99.8%) and methyl-beta-cyclodextrin (MBCD, MW = 1303.3 g/mol) were provided by Sigma-Aldrich (St. Louis, MO, USA). Congo red (CR) (purity > 98.0%) was provided by Tokyo Chemical Industry Co., Ltd., Tokyo, Japan and dimethylformamide (DMF) (p.a. grade, 99.8%) provided by Panreac (Barcelona, Spain) was used as a solvent. Gas permeation experiments and gas solubility measurements were carried out using carbon dioxide (purity ≥ 99.98%) and oxygen (purity ≥ 99.5%), all supplied by Air Liquide (Lisbon, Portugal).

### 2.2. Membrane Preparation

In the present work, all polyurethane-based membranes were prepared with the PUR prepolymer supplied by Fabrires—Produtos Químicos S.A., Lisbon, Portugal without further purification and the chemical structure is represented in Figure S1.

#### 2.2.1. Pure Polyurethane PU Membrane

The PU membrane was synthesized using the PUR prepolymer without requiring the presence of solvent and therefore the total solid to solvent wt.% ratio was of 100/0. The symmetric dense PU membrane was prepared by weighing 10.8 g of the PUR prepolymer and directly casting it onto a glass plate with a 250 µm casting knife at room temperature. The film was further exposed to air at room temperature and continued curing for at least 24 h by atmospheric moisture. Figure S2 shows the possible molecular structure of the PU membrane following the curing of the PUR prepolymer.

#### 2.2.2. Polyurethane-Based Blend Membranes: PU/TRIS, PU/CR, and PU/MBCD

Three polyurethane-based membranes, PU/TRIS, PU/CR, and PU/MBCD were prepared from three casting solutions composed of PUR and TRIS, PUR and CR, PUR and MBCD, respectively. Each of the casting solutions, PU/TRIS, PU/CR, and PU/MBCD were prepared by dissolving PUR and the reagent (TRIS, CR or MBCD) in DMF with a total solid to solvent wt.% ratio of 65/35. The PU to TRIS wt.% ratio was 99.2/0.8 and the PU to CR and PU to MBCD wt.% ratios was 99.6/0.4. The casting solutions were stirred under magnetic stirring for 2 h, cast onto glass plates with a 250 µm casting knife and left to cure at room temperature for 24 h (curing process by atmospheric moisture), rendering PU/TRIS, PU/CR, and PU/MBCD membranes. Table S1 contains information regarding the specific weights of the reagents used to produce the casting solutions od the PU-based blend membranes.

Figure S3 shows the possible molecular structure of the PU/TRIS membrane. TRIS possesses two different reactive functional groups (three primary alcohols and a primary amine), that can both react with PU available NCO groups, leading to structures A (reaction with a primary alcohol) and B (reaction with the primary amine) in Figure S3. However, due the higher nucleophilicity of the amine is expected that this PU/TRIS membrane will be the major product [3]. Figure S4 show the PU/CR membrane chemical structure. Since CR has two amine groups, further reactions may be expected, leading to a crosslinked network. Figure S5 illustrates the structure of the PU/MBCD membrane, were MBCD is a cone-shaped cyclic macromolecule composed by seven units of glucose. 

Similarly to what occurs in the pure PU membrane, in these membranes, the unreacted NCO groups can react with the water molecules present in the atmosphere during the curing stage (following the spreading of the casting solution), but also with themselves forming dimers or trimers [3].

### 2.3. Membrane Characterization

#### 2.3.1. Scanning Electron Microscopy (SEM)

Scanning electron microscopy (JSM-7001F FEG-SEM, JEOL, Tokyo, Japan) was used to visualize the morphology (surface and cross-section) of the prepared membranes. To conduct SEM, samples of the membranes were fractured after freezing in liquid nitrogen, mounted on a stub, and sputter coated with gold. The membranes’ total thickness was measured from SEM cross-section images with ImageJ software. For each membrane, five randomly selected zones from the entire cross-section images were measured and the mean thickness and standard deviation were calculated.

#### 2.3.2. Fourier Transform Infrared Spectroscopy in Attenuated Total Reflection Mode (ATR-FTIR)

The PUR prepolymer, pure TRIS, pure CR, pure MBCD and the active layer surface of the all of the polyurethane-based membranes was analyzed by Fourier transform infrared spectroscopy (FTIR) in attenuated total reflection (ATR) mode. FTIR spectra of five membrane samples of each composition were obtained with a Nicolet Magna IR System 5700 spectrometer (Nicolet Instrument Corp., Madison, WI, USA) using a Golden Gate MKII ATR accessory with a Ge crystal (Graseby Specac, Smyrna; sampling depth: 0.2–1.1 µm at 4000–400 cm^−1^). Each spectrum was obtained by averaging 264 scans with a resolution of 4 cm^−1^. The spectra were transformed to log10(1/R) using OMNIC™ software (Thermo Fisher Scientific, Waltham, MA, USA) and are presented without baseline or smooth corrections.

#### 2.3.3. Mechanical Properties

Uniaxial tensile tests were performed on the pure PU membrane and the PU-based blend membranes, PU/TRIS, PU/CR and PU/MBCD, using an Instron^®^ 5544 universal testing machine (Norwood, MA, USA) coupled with an Instron^®^ standard video extensometer 1 (Norwood, MA, USA), and an Instron^®^ load cell of 100N (Norwood, MA, USA). All the tests were performed on the same day by the same group of researchers to reduce the variability of the testing conditions.

**Figure 2:**
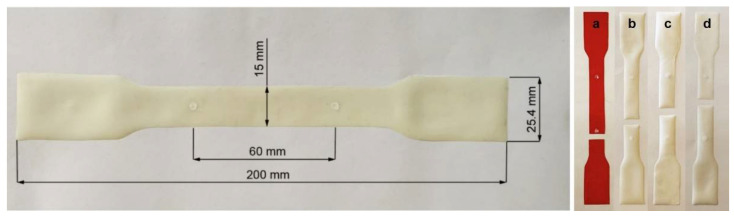
Photograph and dimensions of a tensile test specimen of the PU membrane, and examples of specimens after rupture: PU/CR (**a**), PU (**b**), PU/TRIS (**c**) and PU/MBCD (**d**).

For each composition, multiple specimens were cut considering a dumbbell shape from pre-cast membranes, using a 3D-printed cutting cast (placed always in the same direction to avoid the effects of anisotropy) and scissors. Any specimens exhibiting irregular edges from the cutting process were discarded. Prior to testing, each specimen was marked with two white dots that delimit the gauge length (of 60 mm). These marks act as reference points for the video extensometer, which uses them to calculate the strain along the trial. Figure 2 shows the reference marks and the dimensions used for the tensile specimens. 

The cross-sectional area of each specimen, required to determine the Engineering stress, was assumed to be uniform along the testing region and rectangular (with a width equal to the distance between the casting slits and a thickness equal to the specimen average thickness). The average thickness was calculated considering five measurements taken from different regions of the specimen using a Dexter^®^ digital caliper with 0.01 mm precision (Lezennes, France). 

Before the start of the test, each specimen was carefully placed in between the pneumatic grips of the testing machine. A pre-tension of approximately 0.15 MPa (or 0.2 N) was applied to ensure that the tension was evenly distributed along the cross-section of the specimen. Finally, the pre-tension was released, and the specimens were tested until rupture, at ambient conditions, with a uniform elongation rate of 15 mm/min (0.0041 s^−1^) The evolution of the crosshead displacement, load and video axial strain over time were recorded by a computer using the Instron^®^ Blue Hill version 3 software (Norwood, MA, USA) with an acquisition frequency of 10 Hz. 

The data collected were used to plot the stress–strain curves of each specimen and to compute the different mechanical parameters evaluated in this work (Young’s modulus (E), tangent modulus, yield point, ultimate tensile strength and fracture point) considering in-house routines developed in MATLAB software (MathWorks©, Natick, MA, USA). For the calculation of the Young’s modulus, a linear curve was fitted to the linear region of the stress–strain curve for lower elongations (elastic region) using a least-squares interpolation. A similar approach was used to determine the tangent modulus, defined as the slope of the linear curve for higher elongations. The yield stress/strain of each specimen was determined by selecting the point in which the curve diverged from the linear fitting curve obtained for the Young’s modulus. Additionally, the average values of the tensile properties were calculated from the results obtained for five specimens of each composition. Only specimens which ruptured in the testing area (i.e., the area comprised between the two white dots) were selected for further analysis (see Figure 2).

#### 2.3.4. Gas Permeation

The gas permeability of oxygen and carbon dioxide was measured by the constant volume method using an experimental setup which has been previously described [26]. Briefly, a constant pressure of gas is applied on the feed side of the membrane (surface area 9.62 cm^2^) and the gas flux through the membrane is monitored by measuring the variation of pressure with time in one of two constant volume downstream chambers (volume 13.5 cm^3^ and 26.1 cm^3^) on the permeate side. In permeation measurements using carbon dioxide, the total volume of the receiving chamber was 26.1 cm^3^, while for oxygen, the receiving chamber volume was 13.5 cm^3^. The downstream (permeate) pressure was measured continuously by a vacuum pressure transducer. The change in permeate pressure per unit time was measured by a Paros model 6100A-CE (Redmond, WA, USA) transducer.

CO_2_ and O_2_ permeation curves (pressure vs. time) were obtained for 5 samples of each membrane and at single gas feed pressures (P_0_) ranging between 1.5 and 4.0 bar and at 37.0 ± 0.2 °C. The P(t) curves were used to obtain the volumetric flux of the single gas through each membrane. First, the Ideal Gas Law is used to convert the steady-state region’s slope, $\frac{dp_{p}}{dt}$, of each permeation curve into molar flow, $\frac{dn}{dt}$:

(1) $$\frac{dn}{dt}=\frac{dp_{p}}{dt}\cdot \frac{V_{s}}{RT}$$

where $V_{s}$ is the downstream (receiving) chamber volume (cm^3^), R is the ideal gas constant (0.278 cm^3^ cmHg cm^−3^ (STP) K^−1^), and T is the permeation temperature (K). The Ideal Gas Law was found to be a reasonable approximation to the behavior of gases in the experimental conditions used (low pressure and moderately high temperature). The obtained molar flow is subsequently transformed to volumetric flow, $\frac{dV}{dt}$, at STP conditions:

(2) $$\frac{dV}{dt}=\frac{dn}{dt}\cdot \frac{RT_{STP}}{p_{STP}}$$

where $T_{STP}$ (273.15 K) and $p_{STP}$ (76 cm Hg) are the temperature and pressure in STP conditions. Equation (1) is then substituted in Equation (2), resulting in the following expression:

(3) $$\frac{dV}{dt}=\frac{dp_{p}}{dt}\cdot \frac{V_{s}T_{STP}}{Tp_{STP}}$$

The steady-state volumetric flux, J, of CO_2_ and O_2_ as a function of the TMP for the pure PU, PU-based blend and nanocomposite membranes was obtained and for each membrane and the permeance, perm, defined by the slope of the volumetric flux vs. TMP plot (Equation (4)) was found.

(4) $$perm=\frac{dJ}{d\left(TMP\right)}\left[\frac{{\mathrm{cm}}^{3}\left(\mathrm{STP}\right)}{{\mathrm{cm}}^{2}s\;\mathrm{cmHg}}\right]$$

CO_2_ and O_2_ permeabilities were calculated by:

(5) P=DS=perm×l

where *P* is the permeability coefficient (1 Barrer = 10^−10^ cm^3^(STP) cm/cm^2^ s cmHg), perm is the permeance (cm^3^(STP_)_/cm^2^ s cmHg), *l* is the membrane thickness (cm), *D* is the diffusion coefficient and *S* is the solubility coefficient.

The diffusion coefficient (*D*, cm^2^/s) was calculated using the time-lag method

(6) $$D=\frac{l^{2}}{6\theta }$$

where *θ* is the time lag (*s*) and the intercept obtained from extrapolating the linear region of the experimentally obtained *p*(t) vs. time graphs. The solubility coefficient (*S*) (cm^3^ _STP_/cm^3^.cmHg) was calculated by

(7) $$S=\frac{P}{D}$$

## 3. Results and Discussion

### 3.1. Morphological Analysis by SEM

**Figure 3:**
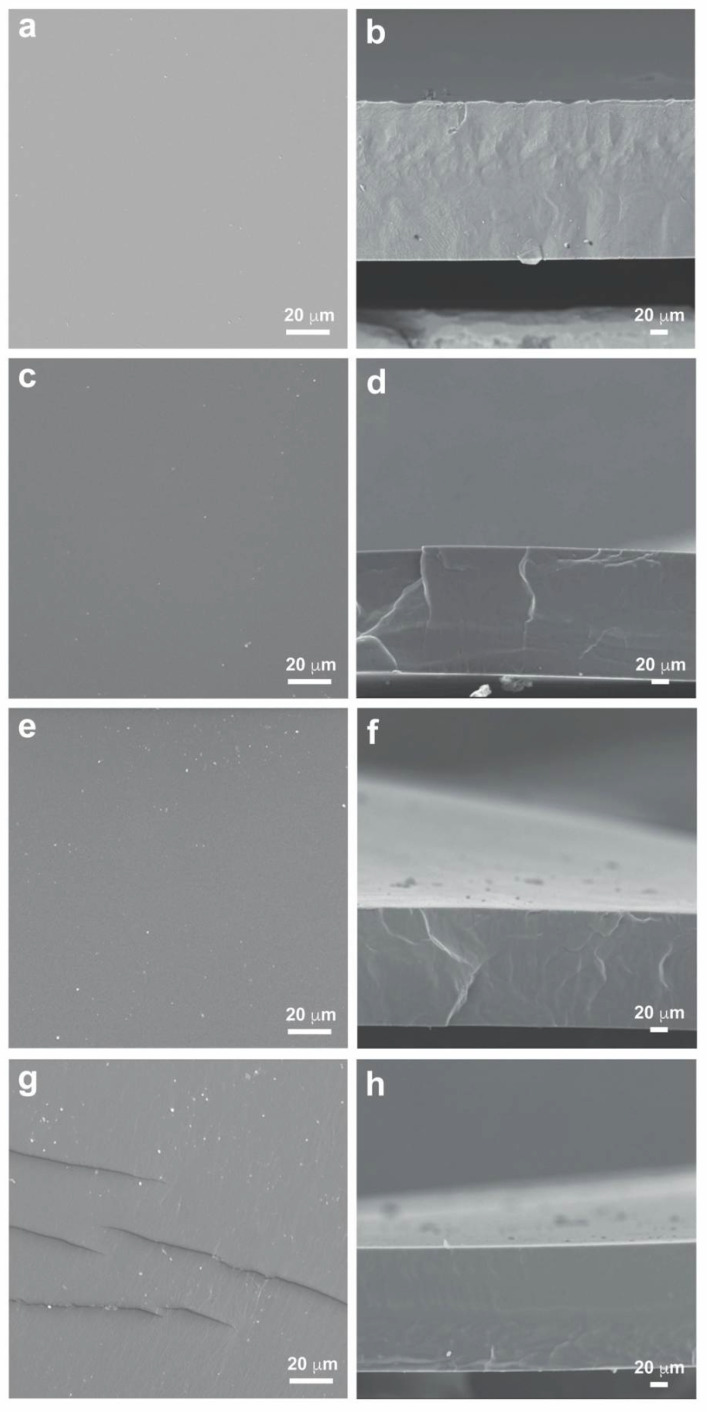
SEM images of the surface and cross of the non-porous symmetric PU-based membranes: (**a**) surface and (**b**) cross-section of the PU membrane, (**c**) surface and (**d**) cross-section of the PU/CR membrane, (**e**) surface and (**f**) cross-section of the of the PU/TRIS membrane, and (**g**) surface and (**h**) cross-section of the PU/MBCD membrane.

Figure 3 shows the images of the surface and cross-sections of the PU and polyurethane-based blend membranes, PU/CR, PU/TRIS and PU/MBCD obtained by SEM. All the membranes exhibit a dense, homogenous morphology with no visible pores. The addition of TRIS, CR and MBCD does not seem to have any influence on membrane morphology when compared to the dense PU membrane, which is synthesized using the same prepolymer PUR. The PU/MBCD membrane contained small fissures sparsely distributed on its surface, which are thought to have originated during the removal of the membrane from the glass plate or from the sputter coating process of the SEM samples.

The total thickness (ℓ) of each membrane was measured on five different points of the cross-section SEM micrographs using the ImageJ software [30]. The average values and respective standard deviations for the PU-based membranes was: 181 ± 0.9 µm for the PU membrane, 149 ± 0.3 µm for the PU/CR membrane, 137 ± 1.0 µm for the PU/TRIS membrane and 144 ± 0.4 µm for the PU/MBCD membrane. The pure PU membrane exhibits the largest thickness suggesting that the introduction of DMF (solvent) as well as the second component (CR, TRIS and MBCD) results in blend membranes with smaller thicknesses. Furthermore, the PU/TRIS membrane presented the smallest thickness.

### 3.2. Chemical Structure by ATR-FTIR

One of the main concerns of this work was to understand the relationships between the membrane structure, the gas permeability and the mechanical properties of the different PU-based membranes. Therefore, ATR-FTIR spectra of four membranes were looked at in greater detail.

**Figure 4:**
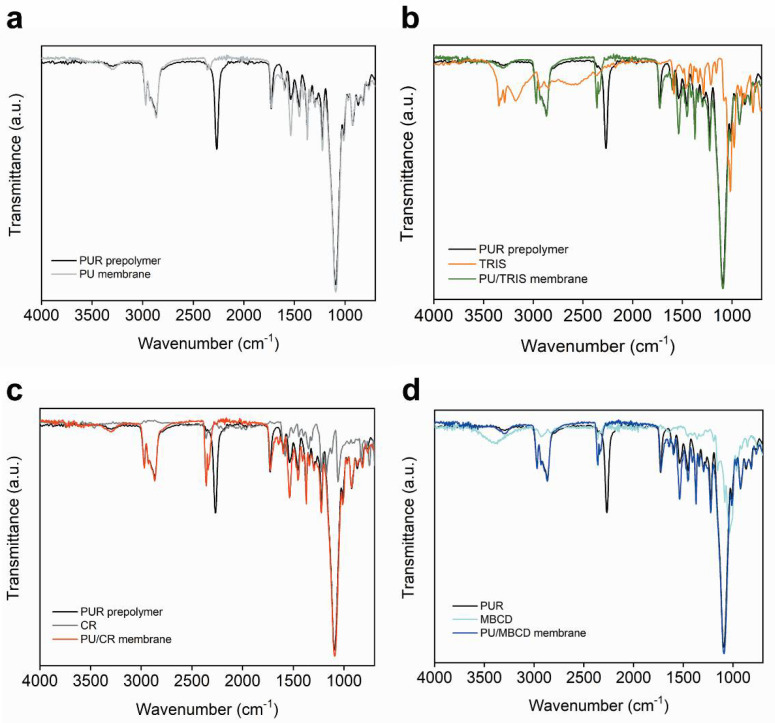
Spectra of each of the PU-based membranes and the pure single components used to prepare them: (**a**) spectra of the PU membrane (grey) and the PUR prepolymer (black); (**b**) spectra of the PU/TRIS membrane (green), PUR prepolymer (black) and TRIS (orange); (**c**) spectra of the PU/CR membrane (red), PUR prepolymer (black) and CR (grey); and (**d**) spectra of the PU/MBCD membrane (dark blue), PUR prepolymer (black) and MBCD (light blue).

Figure 4 shows the spectra of each of the PU, PU/TRIS, PU/CR and PU/MBCD membranes as well as the spectra of the pure single components (PUR prepolymer, TRIS CR and MBCD) used in the casting solutions that were used to prepare the final membranes.

The spectra of the PU (grey line, Figure 4a), PU/TRIS (green line, Figure 4b), PU/CR (red line, Figure 4c) and PU/MBCD (dark blue line, Figure 4d) membranes are mainly characterized by a very weak broad band at approximately 3300 cm^−1^ corresponding to the NH stretch (υNH) [16], a medium intensity peak centered at 2860 cm^−1^ corresponding to the CH_2_ stretching vibration (υCH_2_) [31], a medium intensity peak corresponding to the urethane/urea carbonyl stretch (υC=O) centered at approximately 1725 cm^−1^ [16], a medium intensity band at approximately 1215 cm^−1^ corresponding to the NH (υNH) and CN (υCN) vibrations [31], and a very strong band centered at 1085 cm^−1^ corresponding to the urethane C-O-C asymmetric stretch (υ_as_COC) and the ether aliphatic C-O-C stretch [16].

Figure 4a shows the spectra of the PUR prepolymer and the cured PU membrane. The spectrum of the uncured PUR prepolymer (black) a strong peak centered at 2278 cm^−1^ characteristic of the asymmetric isocyanate stretching mode (υ_as_NCO) [16] is clearly visible. The same peak is not present in the spectrum of the PU membrane, indicating that all of the NCO groups probably reacted with the water molecules present in the atmosphere during the curing process, resulting in a PU dense membrane with urea bonds between molecules of PUR and the terminal NCO groups of were converted in NH_2_ terminal groups. 

Figure 4b shows the spectra of the PUR prepolymer, pure TRIS and the PU/TRIS membrane. The spectrum of pure TRIS (orange) clearly shows the hydroxyl stretching band (υOH) centered at approximately 3350 cm^−1^ [16] and in the spectrum of the uncured PUR prepolymer (black) a peak centered at 2278 cm^−1^ characteristic of the asymmetric isocyanate stretching mode (υ_as_NCO) is clearly visible. In the spectrum of the PU/TRIS membrane (green), the peaks corresponding to (υOH) and (υ_as_NCO) are no longer visible, indicating that probably the isocyanate groups of the uncured PUR prepolymer have reacted with the OH groups of TRIS forming the PU/TRIS membrane, where the urethane/urea carbonyl stretching band (1730 cm^−1^) is clearly visible.

Figure 4c shows the PUR prepolymer, pure CR and the PU/CR membrane. As was seen for the PU/TRIS membrane, in the PU/CR membrane, the band corresponding to the asymmetric isocyanate stretching mode (υ_as_NCO) is no longer visible, indicating that all NCO groups of the PUR prepolymer reacted with the NH_2_ groups present in CR or with water molecules present in the atmosphere during the curing stage, resulting in a PU/CR membrane with the urea group between a molecule of PUR and CR and other urea group between two molecules of PUR.

Figure 4d shows the spectra of the PUR prepolymer, pure MBCD and the PU/MBCD membrane. The spectrum of pure MBCD (light blue) clearly shows the hydroxyl stretching band (υOH) centered at approximately 3350 cm^−1^ and the peak centered at 2278 cm^−1^ in the spectrum of the uncured PUR prepolymer (black), characteristic of υ_as_NCO is also seen. In the spectrum of the PU/MBCD membrane (dark blue) the peak corresponding to (υOH) is reduced and (υ_as_NCO) is no longer visible, indicating that probably all isocyanate groups of the uncured PUR prepolymer have reacted with the OH groups of MBCD forming the PU/MBCD membrane where the urethane/urea carbonyl stretching band (ca. 1730 cm^−1^) is clearly visible.

**The Carbonyl (C=O) Stretching region**

The literature shows that a detailed analysis of the C=O stretching region of segmented polyurethanes can provide information of the hydrogen bonding that occurs between the different functional groups and its relation to the segregation or mixing between hard and soft segments [20,23,25].

The PU, PU/TRIS, PU/CR and PU/MBCD membranes are segmented polyurethanes composed of a polyether soft segment (PPO present in the PUR prepolymer) and polyurethane/urea hard segments which can form hydrogen bonds between each other. The hydrogen bond if formed when a proton of the H atom of the N-H group belonging to the urethane and/or urea functional group is donated to one of the following proton accepting groups: (i) the carbonyl (C=O) group of the urethane or urea functional group—leading to hydrogen bonding between hard segments of the final PU-based blend membrane (phase segregation between hard and soft segments), and (ii) the oxygen atom of the ether functional group (PPO present in the PUR prepolymer)—leading to hydrogen bonding between hard and soft segments of the final U-based blend membrane (mixing between hard and soft segments). The main difference between the hydrogen bonding that occurs with the C=O urea groups and the C=O urethane groups is the capacity of the carbonyl moiety of the urea to interact simultaneously with two NH groups, forming hydrogen bonds in different directions. As a consequence, a three-dimensional structure of hydrogen-bonded urea groups can be formed and is referred to as ordered hydrogen-bonded urea groups [20,25].

**Figure 5:**
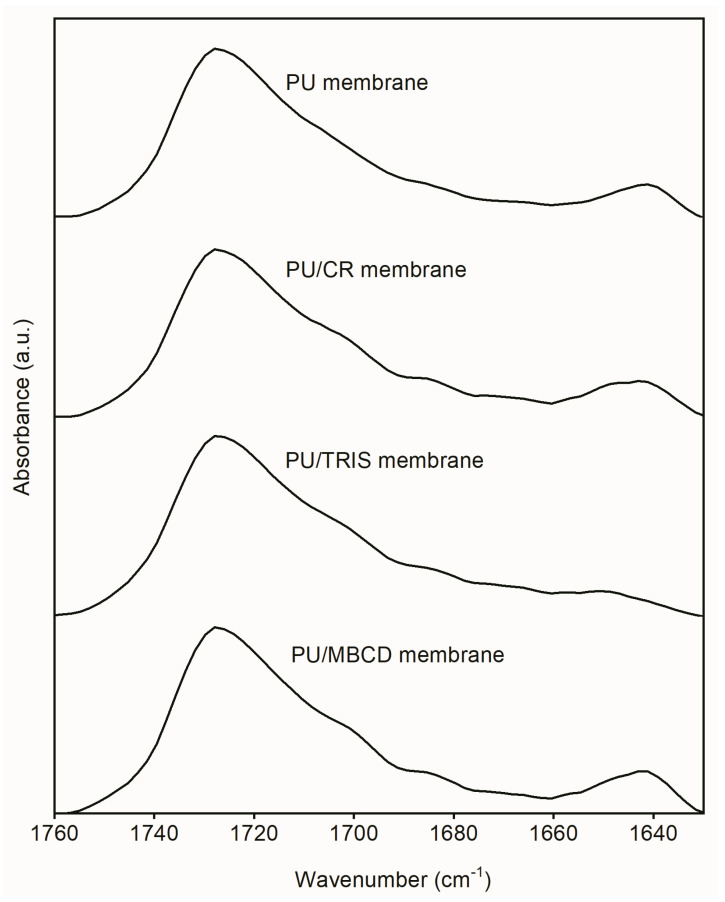
ATR-FTIR spectra of the carbonyl stretching region of the PU, PU/CR and PU/TRIS membranes.

Figure 5 shows the infrared spectra of the carbonyl stretching region (1630–1760 cm^−1^) of the PU, PU/TRIS and PU/CR membranes. All spectra are characterized by a strong band centered at approximately 1730 cm^−1^, corresponding to free C=O urethane groups, and a shoulder at approximately 1714 cm^−1^, which is assigned to urethane groups involved in hydrogen bonds. Two weak shoulders can be identified in the region 1690–1700 and 1660–1675 cm^−1^, attributed to free urea carbonyl and disordered hydrogen-bonded urea carbonyl, respectively. The band centered at 1640 cm^−1^ corresponds to the hydrogen-bonded ordered urea carbonyl.

**Figure 6:**
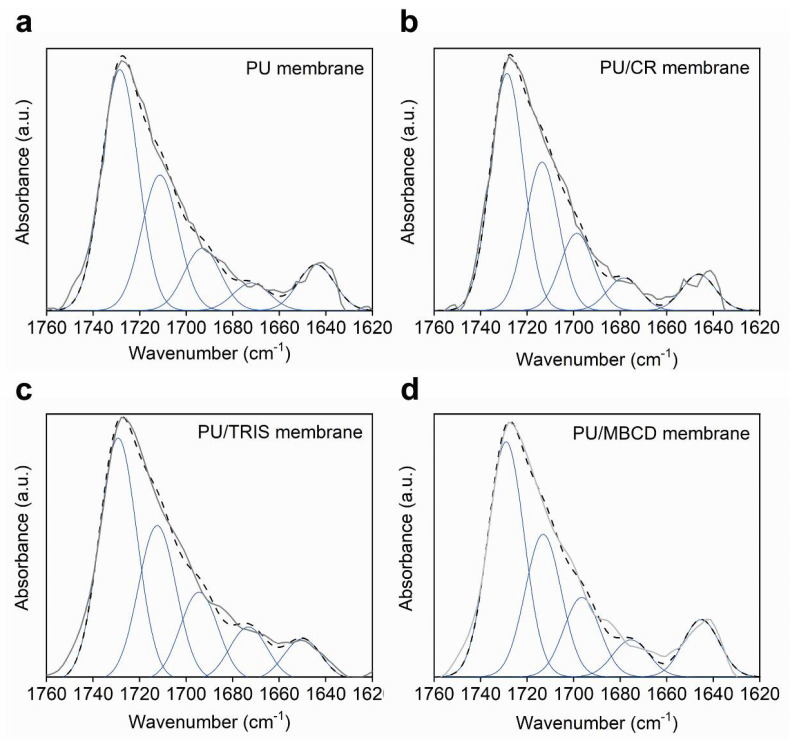
Curve-fitting decomposition of the carbonyl stretching band of the spectra obtained from the (**a**) PU, (**b**) PU/CR, (**c**) PU/TRIS and (**d**) PU/MBCD membranes. ATR-FTIR original spectra are shown by the thick grey line and the simulated band by the dashed line.

The C=O stretching vibration band was decomposed by curve fitting (Levenberg-Marquardt algorithm) between 1630 and 1760 cm^−1^. Five Gaussian bands corresponding to free and hydrogen-bonded urethane carbonyl groups and free and hydrogen-bonded disordered and ordered carbonyl urea groups were employed in the fitting procedure. The starting position of the bands used in the fitting were obtained from the second-derivative spectrum of the C=O band and during the fitting procedure the peaks were allowed to shift freely while fixing the width at half-height. Figure 6 shows the simulation and deconvolution of the bands for the PU, PU/CR, PU/TRIS, and PU/MBCD membranes and Table 1 shows the frequency (υ) and relative areas A (%) of the deconvoluted bands.

The results obtained by curve-fitting procedures give quantitative and relative information of the number of different species in each membrane. The frequency of the free and hydrogen-bonded urethane carbonyl stretching vibrations remained essentially constant for all membranes, with values of approximately 1729 and 1713 cm^−1^, respectively. In all membranes the area corresponding to the free urethane carbonyl groups is higher than that one of the bonded urethane C=O group. The coefficient of the hydrogen-bonded C=O group is much higher than that of the non-hydrogen-bonded C=O group [32], therefore, we can conclude that for all of the membranes most urethane C=O groups are not involved in hydrogen bonds. The PU membrane presents a larger number of free urethane C=O groups when compared to the rest of the membranes and the PU/CR membrane presents the highest number of H-bonded urethane C=O groups.

The frequency of the free urea carbonyl stretching vibrations were centered between 1694 and 1699 cm^−1^ and the disordered and ordered hydrogen-bonded varied between 1672 and 1678 cm^−1^ and 1645 and 1650 cm^−1^, respectively. The PU/CR membrane presents the highest number of free urea C=O groups while the PU/TRIS presented the highest number of disordered H-bonded urea C=O groups and the PU/MBCD membrane the highest number of ordered H-bonded urea C=O groups.

The ratio (%) of H-bonded to free urethane/urea carbonyl groups for the PU membrane was 39:61, indicating that most C=O groups are not hydrogen bonded to other functional groups which may be an indication phase segregation. The bonded:free ratio of the PU/CR membrane was 55:45, indicating that the number of urethane and urea groups participating in hydrogen bonds is close to the number of free C=O groups which may indicate mixing between different segments of the PU-based membrane. The bonded:free ratio of the PU/TRIS and PU/MBCS membranes is 57:43, indicating that the different segments in these membranes show a higher degree of mixing than the PU membrane but lower than the one found for the PU/CR membrane.

Among all of the membranes analyzed, the PU membrane showed the highest degree of free carbonyl urethane/urea groups (belonging to hard segments) and therefore is expected to have the lowest extent of mixing between hard segments and soft segments and, consequently, to have the largest phase separation. In contrast, the PU/CR membrane showed the highest degree of bonded carbonyl urethane/urea groups (belonging to hard segments) and therefore is expected to have the highest extent of mixing between hard segments and soft segments and, consequently, to have the lowest phase separation.

### 3.3. Mechanical Properties

To compare the mechanical properties of the PU, PU/TRIS, PU/CR and PU/MBCD membranes, uniaxial tensile tests were performed on all membranes. All of the test specimens included in the analysis ruptured in the gauge length region, i.e., specimens in which the failure occurred on the grips or on the curvature region were discarded from the trials. All specimens presented a ductile fracture with the failure line perpendicular to the direction of the applied load and no visible failures were observed in other regions of the test specimen (see Figure 2).

**Figure 7:**
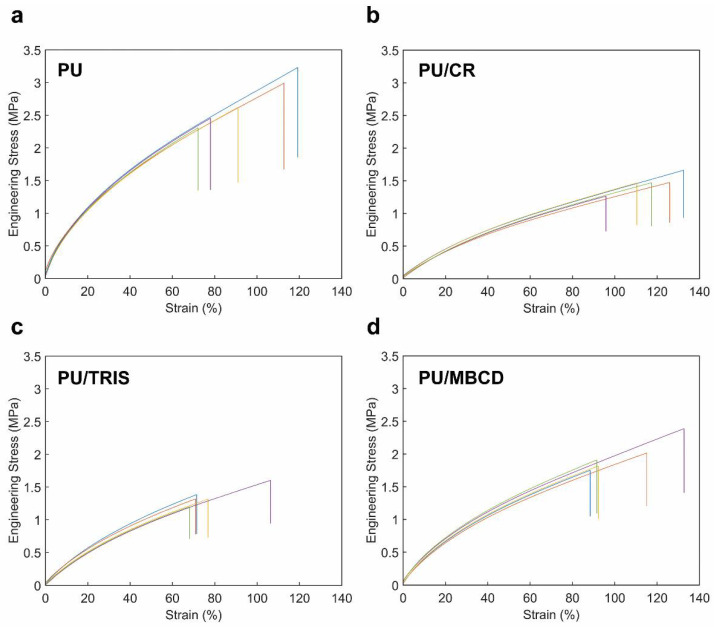
Stress–strain curves obtained for the pure polyurethane and polyurethane blend membranes: (**a**) PU, (**b**) PU/CR, (**c**) PU/TRIS and (**d**) PU/MBCD. Multiple specimens are represented by different colors for each membrane.

Figure 7 shows the engineering stress–strain relationships obtained for five selected specimens of each of the specimens of the PU, PU/TRIS, PU/CR and PU/MBCD membranes. All membranes display similar stress–strain behaviors, typical of elastomers, with an initial linear region (small elongations—elastic domain), followed by a region characterized by a decrease in the curve slope with the increase in the strain, finally reaching a steady slope region (tangent modulus) for higher elongations rates (strain >~20%). This behavior can be explained by the disentanglement of the polymer chains, through the break of the covalent crosslinks and hydrogen bonds, and then their stretching until achieving the failure point [33,34] despite accomplishing values of elongation at break of approximately 79% to 116%, the specimens partially recovered to a configuration similar to the one they had before the tensile tests (see Figure 2). This issue indicates that only a small part of the strain observed in the stress–strain curves is explained by the existence of plastic deformation of the polymeric chains. The structural morphology of the PU can also explain the restoring of the configuration observed after unloading the specimen. The crosslinks and hydrogen bonds between chains are restored and the membrane returns to a configuration similar to the original one.

Table 2 shows the average values of the Young’s modulus (E), tangent modulus, ultimate tensile strength (UTS) and elongation at break obtained for each type of membrane.

Moreover, the analysis of the stress–strain curves allows to observe similar properties for specimens of the same group, presenting similar Young’s moduli, yield points and tangent moduli. A higher variability is only observed in the failure region. These results support the idea that membranes prepared in the same bath are relatively homogeneous, not presenting significant differences in terms of tensile mechanical properties. 

The Young’s modulus, given by the initial slope of the stress–strain curve, is a measure of the material’s stiffness. Generally, elastomers such as polyurethanes are said to be soft materials which sustain large deformations under relatively small forces, as opposed to glassy polymers which are stiffer and require large forces to reach small deformations. These ideas are supported by the values obtained for the Young’s modulus, which ranged from 2.28 MPa in PU/CR to 7.99 MPa in PU pure membranes. Similarly, the values measured for the tangent modulus ranged from 0.95 MPa to 2.09 MPa. When the blended variations are compared with the pure PU membranes, all presented a reduction in the Young’s and tangent modulus. The higher reduction was observed in the PU/CR membranes (E: −71.5%, tangent modulus: −54.5%), followed by the PU/TRIS (E: −62.0%, tangent modulus: −36.4%). The PU/MBCD composition presented an intermediate E and tangent modulus value, but still significantly lower than the pure PU membrane (E: −48.1%, tangent modulus: −34.0%).

The ultimate tensile strength (UTS), or simply tensile strength, is the maximum stress that can be applied to a material. In this case, it coincides with the stress at failure, as the maximum tension occurred before the specimen break. The UTS results were all within the same order of magnitude, and once again, the PU membrane presented the highest tensile strength, followed by the PU/MBCD (−27.2%) and finally by the PU/CR (−46.0%) and PU/TRIS (−50.0%) compositions, exhibiting similar lower values. 

Another important parameter that can be obtained from the stress–strain diagram is the elongation at break, or the amount of strain (%) under which the material ruptures. The highest average value was found for the PU/CR composition at 116.4%. The PU and PU/MBCD membranes ruptured at intermediate elongations of 94.7% and 104.0%, respectively. Finally, the PU/TRIS specimens ruptured at a significantly lower average elongation of 78.7%. The relatively high elongations observed for the tested membranes are coherent with the behavior observed in elastomers, in which the PU membranes inserts. 

The differences observed between the pure and blended PU results indicates that the addition of the three different organic additives used in this work affect the mechanical properties of the membranes. In particular, the introduction of the TRIS, MBCD and CR decreased the stiffness of the membranes both in elastic and plastic domain, meaning that lower forces/pressures are required to achieve a given strain level. These differences can be explained by the different HS/SS ratios on the three compositions and the use of solvent. The PU pure membrane is characterized by a larger HS/SS ratio, which justifies the higher values of Young’s and tangent modulus and UTS. Other factor possibly contributing towards the reduced moduli and UTS values observed in the PU-based blend membranes is the preparation method, since their casting solutions undergo two hours of agitation with large proportions of solvent, while the PU membrane is cast directly from the prepolymer.

The differences between the mechanical properties of the PU-based membranes can also be explained by the degree of mixing between the different phases composed of SSs and HSs. According to ATR-FTIR spectra, PU/CR showed the highest degree of mixing between hard and soft segments, explaining the lowest values of Young’s and tangent moduli and UTS. In turn, the pure PU membrane exhibited the highest degree of phase segregation between hard and soft segments which correlates to the highest values of Young’s and tangent moduli and UTS. ATR-FTIR spectra of the PU/TRIS and PU/MBCD membranes reveal similar degrees of mixing between soft and hard segments which is lower than the one seen for the PU/CR membrane but much higher than is seen for the PU membrane. In accordance, the values of Young’s and tangent moduli for the PU/TRIS and PU/MBCD membranes are higher than those found for the PU/CR membrane but lower than those of the PU membrane. As expected, the UTS value of the PU/MBCD membrane is higher than the value found for the PU/CR membrane and lower than the one found for the pure PU membrane. The value of UTS of the PU/TRIS membrane is lower than the value of the PU/CR membrane, which is unexpected and may be explained by a different micro-structural arrangement or the existence of micro-failures in the membranes. This reduction in the value of UTS also traduced in a clear reduction in the elongation rate at failure in this type of membranes.

ATR-FTIR spectra and mechanical tests suggest that the introduction of the three organic additives, CR, TRIS and MBCD, increased the mobility of the molecular structure of the PU/CR, PU/TRIS and PU/MBCD membranes when compared to the pure PU membrane and consequently reduced its stiffness.

It is important to note that despite reducing the tensile mechanical properties of PU membranes, the addition of the three organic additives does not change drastically their expected mechanical function. If the PU-based blend membranes are intended to be used in gas permeation applications which operate at low pressures (<1 bar) such as blood oxygenation devices, the membranes will not be subjected to load/pressures so high that the lower moduli and UTS values influence their function. When compared with other families of membranes used for gas permeation in hemodialysis purposes (e.g., polyethersulfone (PES), polyvinylidene fluoride (PVDF) or polypropylene), PU-based membranes present lower UTS values [35,36,37]. However, it should be noted that, although lower, the UTS values obtained in the present work are in the same order of magnitude of the ones observed in PES-based membranes [35], which are usually referred in literature as having good to excellent mechanical properties [38].

A comparative analysis of the results obtained with literature [39] enables to find values of the same order. In particular, when the values of the Young’s modulus and tangent modulus of the PU pure membranes are compared with the membranes with the near HS/SS ratio (PU8) and strain rate (0.005 s^−1^), a reduction of 31.7% and 7.9% is observed, respectively. A decrease in the strain at failure is also observed in our case. These differences, both in the strain and moduli values, can be explained by different factors. The first one is related with the differences on the composition and materials used in both works. In [39], the strain was calculated using the crosshead displacement, which can introduce experimental errors related with clearances in the different components of the tensile machine and specimens slip. In this work, a non-contacting video extensometer was used to measure directly the strain in the membranes, avoiding some of the experimental errors addressed before. Lastly, the near strain ratio used in [39] (0.005 s^−1^) is slightly higher than the one used in this work (0.004 s^−1^). Knowing that PU membranes present a viscoelastic behavior, the use of a higher strain rate results in a higher Young’s modulus and UTS, as also shown in [30]. It is important to note that the use of a low elongation rate in this work had in consideration the possible application of this type of membranes in gas permeation devices, where the fluid flow is almost constant. The use of higher strain rates could overestimate the tensile properties of the membranes, as a result of their viscoelastic properties [39], achieving values that do not present a direct relation with in-service conditions.

### 3.4. Gas Permeation

**Figure 8:**
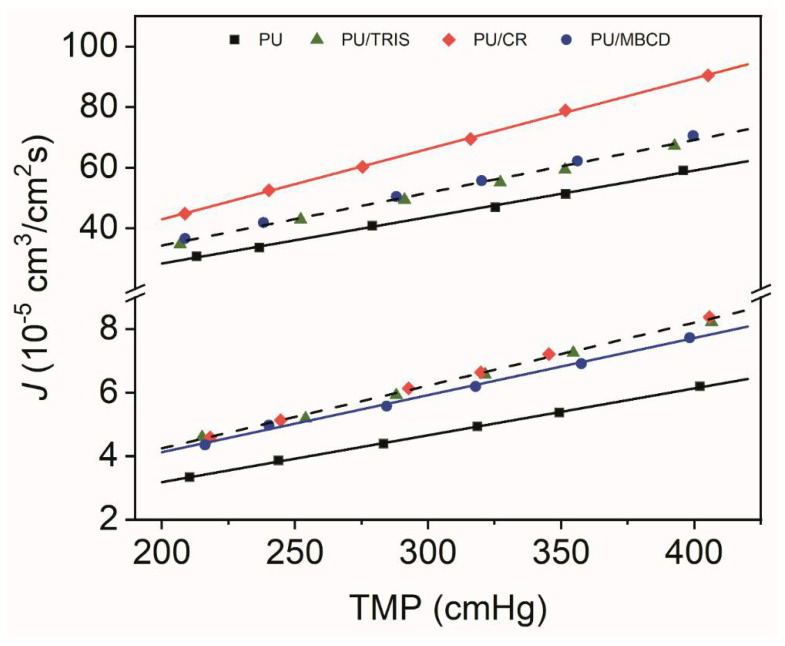
O_2_ (lower group of points—below 20 × 10^−^^5^ cm^3^/cm^2^ s) and CO_2_ (higher group of points—above 20 × 10^−^^5^ cm^3^/cm^2^ s) volumetric fluxes (*J*) vs. the transmembrane pressure (TMP) for the PU (black square), PU/TRIS (green triangle), PU/MBCD (blue circle), and PU/CR (red diamond) membranes. Lines (straight-line fit using the method of least squares) for CO_2_ permeation flux vs. TMP: PU membrane—solid black line, PU/CR—solid red line and PU/TRIS and PU/MBCD membrane in dashed black line. For O_2_ permeation flux vs. TMP: PU membrane—solid black line, PU/MBCD—solid blue line, and PU/TRIS and PU/CR membranes in dashed black.

Figure 8 shows the volumetric flux (J) of O_2_, and CO_2_ as a function of the transmembrane pressure (TMP), which is defined as the difference between the feed pressure and the initial permeate pressure, for the PU, PU/CR, PU/TRIS and PU/MBCD membranes. As can be seen, the steady-state volumetric fluxes J increase linearly as a function of TMP in each series of measurements. Moreover, the values of *J* for CO_2_ are one order of magnitude higher than for CO_2_. For both gases, lower fluxes were observed for the PU membrane. In terms of CO_2_ permeation fluxes, it is obvious that the PU/CR membrane presents higher fluxes than the PU/TRIS and PU/MBCD membranes whose points follow the same trend (dashed line). In terms of O_2_ fluxes it is evident that the PU membrane presents a lower permeation flux compared to the other three membranes. The O_2_ flux of the PU/MBCD membrane is slightly lower than the fluxes found for the PU/CR and PU/TRIS membranes which present the highest O_2_ permeation flux vs. TMP trend (dashed line). Permeation flux values depend on the membrane thickness and therefore, to compare the gas transport properties of the membranes the total thickness of each membrane must be taken into consideration. Table 3 shows the permeances, obtained from the slopes of the J vs. TMP plots depicted in Figure 8, and the permeability coefficients (P), obtained by Equation (5) using the values of membrane thickness, *l*, towards O_2_ and CO_2_ for the PU, PU/CR, PU/TRIS and PU/MBCD membranes.

The results show that the CO_2_ permeances and permeability coefficients are approximately 10 times higher than for O_2_. In terms of CO_2_, the Permeability coefficient of the PU/CR is the highest with a value of 347 Barrer followed by the PU, PU/MBCD and PU/TRIS membranes with values of 278, 251, and 239, respectively. When compared to the membrane composed purely of PU, only the PU/CR blend membrane revealed an increase in CO_2_ permeability. In terms of O_2_ permeability, the PU/MBCD was the membrane with the lowest value, 26 Barrer, followed closely by the PU/ and PU/TRIS membranes, 27 Barrer, and the PU/CR membrane exhibited the highest value of O_2_ permeability, 30 Barrer. The introduction of CR into with the PUR prepolymer lead to a PU/CR blend membrane with higher CO_2_ and O_2_ permeability than the pure PU membrane. The PU/TRIS and PU/MBCD blend membranes exhibited equal or lower CO_2_ and O_2_ permeabilities as the PU membrane indicating that the addition of pure TRIS or MBCD to the PUR casting solutions did not result in enhanced gas transport when compared to the pure PU membrane.

Comparison of the gas transport, mechanical properties and the chemical structure (obtained in the ATR-FTIR) of the PU-based membranes leads to a clear correlation between CO_2_/O_2_ permeation, Young modulus and tensile strength as well as the degree of phase segregation between the different segments. The PU membrane, which presents the lowest O_2_ and CO_2_ permeabilities is also the membrane which exhibits the highest degree of phase segregation (lowest degree of mixing) and the highest Young’s modulus (E) and tensile strength (UTS). 

When compared to permeability coefficients of membranes used in current BOs, the P(CO_2_) values obtained for the PU-based membranes are higher than the ones claimed for the PMP membranes currently used in BOs (90 Barrer). The P(O_2_) values obtained for the PU/CR membrane is equal to the value claimed for PMP membranes (30 Barrer) [40]. 

The diffusion (D) and solubility (S) coefficients of each gas in the PU, PU/MBCD and PU/TRIS membranes were estimated from the permeation curves (Pp vs. time) by the time lag method. The diffusion coefficient (D) is calculated using Equation (6), and the solubility coefficient (S), is then obtained from the product P = DS (Equation (7)), using the values of the permeability coefficients (P) determined by Equation (5) and presented in Table 3.

Table 4 shows the values of t_lag_, D and S obtained towards CO_2_, and O_2_ for the PU, PU/MBCD and PU/TRIS membranes.

Regarding the diffusion coefficients, the values for the PU, PU/TRIS and PU/MBCD membranes are always in the order D(CO_2_) < D(O_2_). In contrast, for the PU/CR membrane the diffusion coefficient for CO_2_, 2.98 × 10^−6^ cm^2^/s is higher than the one for O_2_, 2.52 × 10^−6^ cm^2^/s. The kinetic diameters of CO_2_ and O_2_ are 3.30 and 3.46 Å [41] making them very similar in size so the fact that D(CO_2_) is smaller than D(O_2_) for all of the membranes (with the exception of PU/CR) cannot be explained by the size of the gas molecules. The smaller values of D(CO_2_) may be due to its polar character, that promotes polar interactions with the polymer matrix of the PU, PU/TRIS and PU/MBCD membranes which may hamper its mobility by diffusion and in contrast, promote O_2_ mobility. Comparing the O_2_ diffusion coefficients of the different membranes the highest O_2_ diffusion coefficient was found for the PU/CR membrane, followed by the PU/PMBCD membrane and the lowest values were for the PU/TRIS and PU membranes.

The CO_2_ solubility coefficient for all of the membranes is always much larger (at least 9 times higher) than the O_2_ solubility. It has been proposed that the solubility is related to the gas boiling point or critical temperature of the gas [39]. The boiling point of CO_2_ and O_2_ is −78.5 °C and −183 °C, respectively [40,41]. CO_2_ has the highest boiling point, which correlates to its high solubility in the membrane.

The solubility of CO_2_ is highest for the PU/MBCD membrane and lowest for the PU/CR membrane. This is in contrast to the CO_2_ permeability of these membranes with the PU/CR membrane presenting the highest value (347 Barrer) and PU/MBCD presenting the second lowest value (251 Barrer). Furthermore, the O_2_ solubility for all membranes was similar ranging from 11 to 13 × 10^−4^ cm^3^_STP_/cm^3^cmHg.

A joint analysis of the coefficients in Table 2 and Table 3 clearly shows that the permeation of O_2_ through the PU, PU/TRIS, PU/CR and PUI/MBCD membranes is a diffusion-controlled process. Although O_2_ presents slightly different diffusion coefficients for all membranes, it is the disparity in their diffusion coefficients that induces the differences observed in the O_2_ permeabilities. The same conclusion is reached when analyzing the P, S and D coefficients for CO_2_ through the different membranes. The PU/CR membrane is the membrane that presents by far the highest CO_2_ permeability which correlates to the highest CO_2_ diffusion coefficient and the lowest CO_2_ solubility coefficient of all of the membranes.

## 4. Conclusions

Three novel PU-based dense symmetric membranes, PU/CR, PU/TRIS and PU/MBCD, were prepared by the solvent evaporation method. A control dense symmetric pure PU membrane was also prepared by casting the PUR prepolymer onto a glass plate and leaving it to cure at room temperature. All the membranes contain HSs composed of urethane and urea groups which are dispersed in a matrix of SS blocks composed of a sequence of PPO molecules. The introduction of a second component was found to influence the final phase segregation, gas permeation and mechanical properties when compared to the pure PU membrane. 

SEM images of the surface and cross-sections of the membranes reveal a dense, homogenous morphology with no visible pores and measurements of the total membrane thickness reveal that it decreased in the order PU, PU/CR, PU/MBCD and PU/TRIS membranes. 

ATR-FTIR analysis of the carbonyl stretching area reveals that the PU membrane showed the lowest extent of mixing between hard segments and soft segments and, consequently, have the largest phase separation. In contrast, the PU/CR membrane showed the highest extent of mixing and, consequently, to have the lowest phase separation.

Gas permeation measurements by the constant volume method reveal that the CO_2_ permeability coefficient was highest for the PU/CR membrane (347 Barrer) followed by the PU membrane (278 Barrer), the PU/TRIS membrane (251 Barrer) and finally by the PU/TRIS membrane. 

The O_2_ permeability coefficients of all membranes is 10- to 30-fold lower than the CO_2_ permeabilities (ranging between 26 and 30 Barrer) and was highest for the PU/CR membrane. Comparing the PU and PU/CR membranes, there is clear indication that the CO_2_ and O_2_ permeabilities increase with the increase in mixing between hard and soft segments.

Evaluation of the Young’s modulus, tangent modulus and UTS revealed differences in the mechanical properties of the PU-based membrane which correlate to the degree of phase segregation present in the different membranes. 

Our data suggest that the introduction of organic additives, such as CR, TRIS and MBCD, increases the mobility of the molecular structure of the membranes, when compared to the pure PU membrane, thus being a simple and straightforward strategy for the modulation of polyurethane-based membranes.

## Figures and Tables

**Table 1:** Frequency (υ) and relative area (A) of the regions of the C=O stretching band.

	Urethane				Urea					
	Free		H-Bonded		Free		Disordered H-Bonded		Ordered H-Bonded	
Membrane	υ (cm^−^^1^)	A (%)	υ (cm^−^^1^)	A (%)	υ (cm^−^^1^)	A (%)	υ (cm^−^^1^)	A (%)	υ (cm^−^^1^)	A (%)
PU	1728	46	1712	25	1694	15	1672	5	1645	9
PU/CR	1729	39	1714	32	1699	16	1678	7	1646	6
PU/TRIS	1729	42	1712	27	1694	15	1673	9	1650	7
PU/MBCD	1729	43	1713	26	1696	14	1675	7	1645	10

**Table 2:** Mechanical properties (Young’s modulus, tangent modulus, UTS and elongation at break) of the pure polyurethane and polyurethane blend membranes, obtained from the tensile tests.

Membrane	E (MPa)	Tangent Modulus (MPa)	UTS (MPa)	Elongation at Break (%)
PU	7.99 ± 0.38	2.09 ± 0.19	2.72 ± 0.38	94.65 ± 20.80
PU/CR	2.28 ± 0.08	0.95 ± 0.05	1.47 ± 0.14	116.35 ± 14.29
PU/TRIS	3.04 ± 0.25	1.33 ± 0.13	1.36 ± 0.15	78.70 ± 15.79
PU/MBCD	4.15 ± 0.13	1.38 ± 0.10	1.98 ± 0.25	103.99 ± 19.28

**Table 3:** CO_2_ and O_2_ permeances and permeability coefficient (P) for the non-porous symmetric membranes PU, PU/TRIS, PU/MBCD and PU/CR membranes.

		CO_2_		O_2_	
Membrane	l (µm)	Permeance ^a^	P (Barrer ^b^)	Permeance ^a^	P (Barrer ^b^)
PU	181 ± 0.9	0.1537	278	0.0148	27
PU/CR	149 ± 0.3	0.2328	347	0.0198	30
PU/TRIS	137 ± 1.0	0.1743	239	0.0198	27
PU/MBCD	144 ± 0.4	0.1743	251	0.0180	26

^a^ 10^−^^5^ cm^3^_STP_/cm^2^scmHg. ^b^ 10^−10^ cm^3^_STP_ cm/cm^2^scmHg.

**Table 4:** Time lag (t_lag_) values, diffusion coefficients (D), and solubility coefficients (S) obtained for the permeation of O_2_ and CO_2_ through the PU, PU/CR, PU/TRIS and PU/MBCD membranes.

	CO_2_			O_2_		
Membrane	t_lag_ (s)	D (10^−6^ cm^2^/s)	S (10^−4^ cm^3^_STP_/cm^3^cmHg)	t_lag_ (s)	D (10^−6^ cm^2^/s)	S (10^−4^ cm^3^/cm^3^cmHg)
PU	32.7	1.70	167	27.4	1.99	13
PU/CR	12.4	2.98	116	14.7	2.52	12
PU/TRIS	22.2	1.41	169	14.7	2.13	13
PU/MBCD	25.6	1.35	186	15.3	2.26	11

## Data Availability

Not applicable.

## Supplementary Materials

The following supporting information can be downloaded at: https://www.mdpi.com/article/10.3390/membranes12090826/s1, Figure S1: Chemical structure of PUR, a PPO-based polyurethane prepolymer (*n* = 20); Figure S2: Probable chemical structure of the PU membranes (*n* = 20); Figure S3: Possible chemical structures for the PU/TRIS membrane: reaction with the amine group (A) and reaction with an alcohol group (B); Figure S4: Chemical structure of the PU/CR membrane; Figure S5: Chemical structure of the PU/MBCD membrane; Table S1: Casting solution compositions of the PU/TRIS, PU/CR and PU/MBCD membranes.
