# Supplementary material for: Gas Permeability and Mechanical Properties of Polyurethane-Based Membranes for Blood Oxygenators
Source: Membranes (Basel). 2022 Aug 24;12(9):826. doi: 10.3390/membranes12090826 (PMC9502098; doi:10.3390/membranes12090826)
Supplement: Supplementary file 1 [file membranes-12-00826-s001.zip › membranes-1866412-supplementary.pdf]

Supplementary Materials:

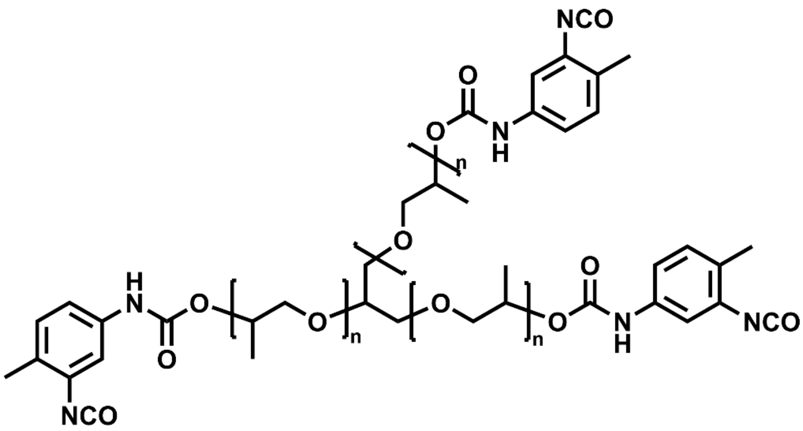

Figure S1. Chemical structure of PUR, a PPO-based polyurethane prepolymer (n= 20).

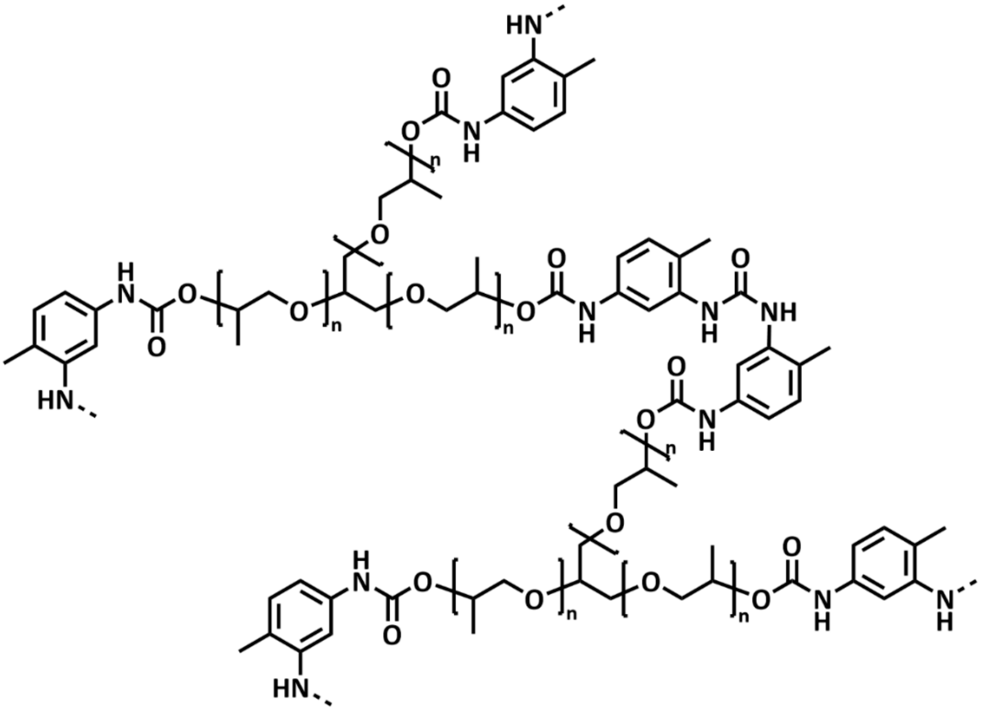

Figure S2. Probable chemical structure of the PU membranes (n= 20).

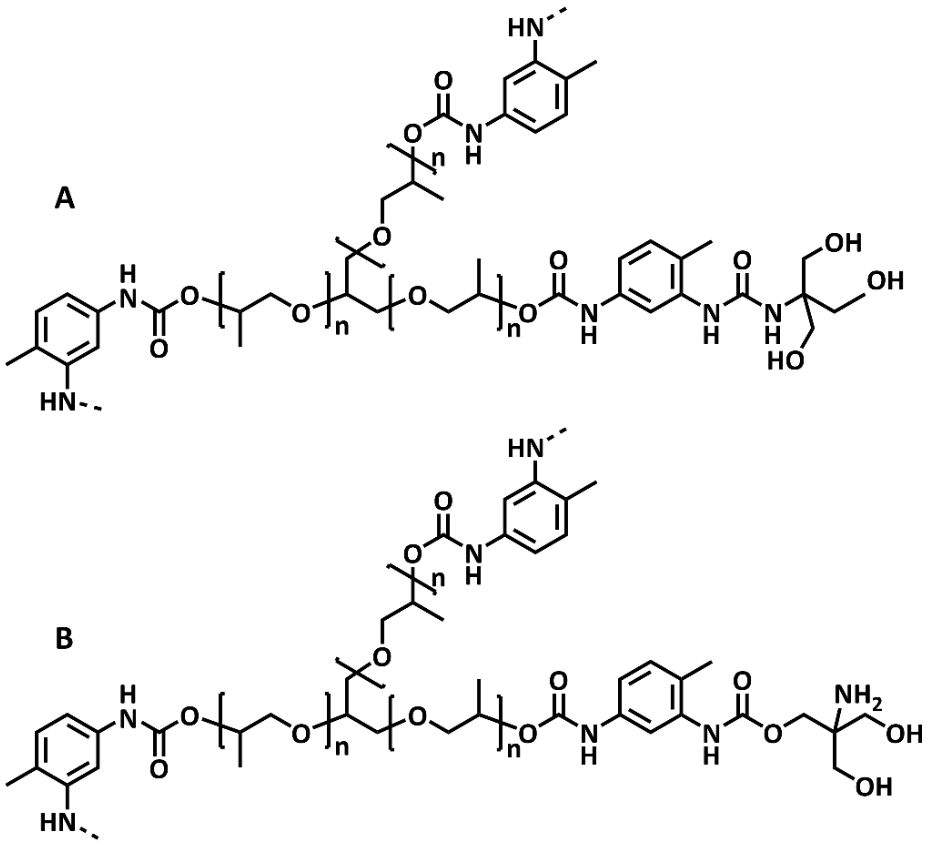

Figure S3. Possible chemical structures for the PU/TRIS membrane: reaction with the amine group (A) and reaction with an alcohol group (B).

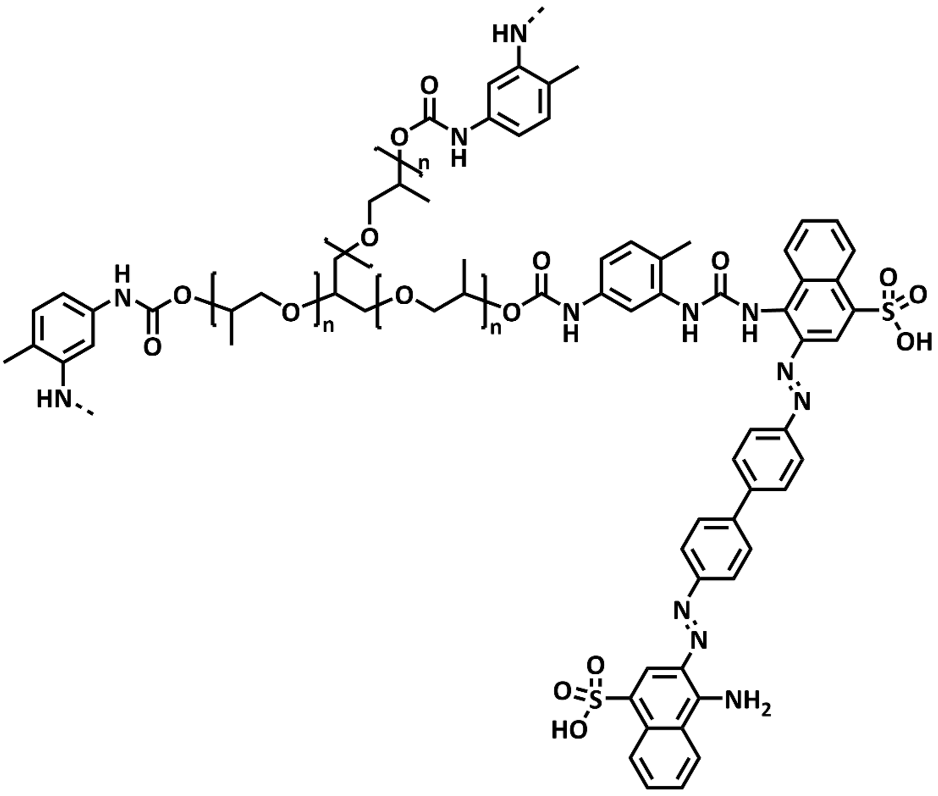

Figure S4. Chemical structure of the PU/CR membrane.

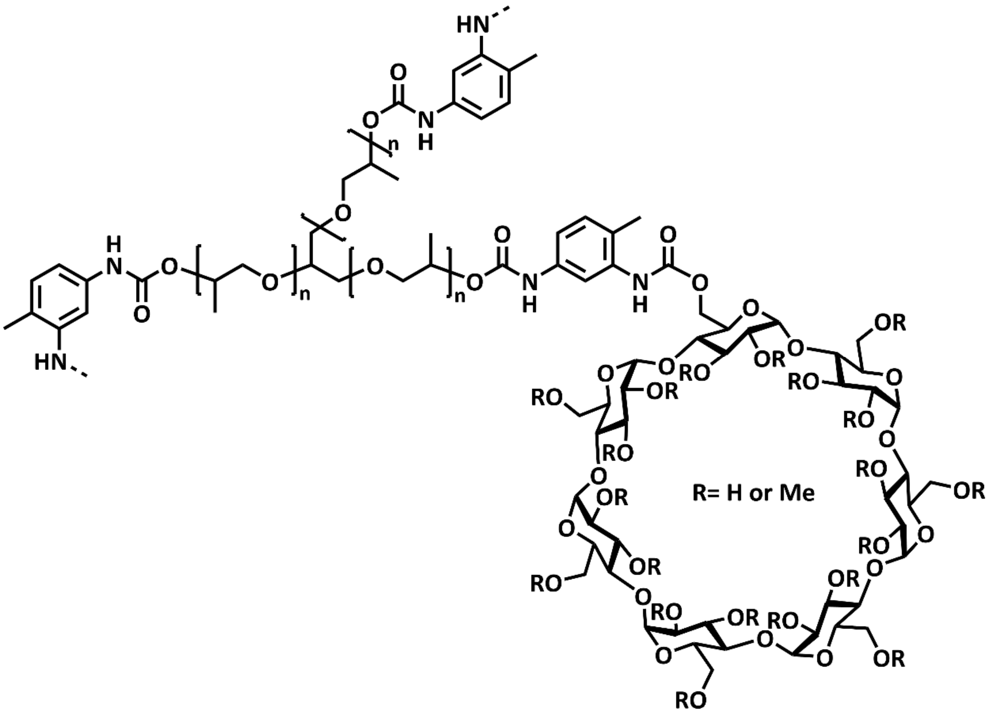

Figure S5. Chemical structure of the PU/MBCD membrane.

Table S1. Casting solution compositions of the PU/TRIS, PU/CR and PU/MBCD membranes.

	PUR (g)	TRIS (g)	CR (g)	MBCD (g)	DMF (mL)
**PU/TRIS**	9.92	0.08	-	-	4.54
**PU/CR**	9.96	-	0.04	-	4.54
**PU/MBCD**	9.96	-	-	0.04	4.54
